# Evaluation of T cell responses in healing and nonhealing leishmaniasis reveals differences in T helper cell polarization *ex vivo* and *in vitro*

**DOI:** 10.1111/j.1365-3024.2009.01094.x

**Published:** 2009-04

**Authors:** B-S CHOI, P KROPF

**Affiliations:** Department of Immunology, Faculty of Medicine, Imperial College LondonUK

**Keywords:** *cell proliferation*, *T cells*, *Th1/Th2 cells*

## Abstract

*Experimental leishmaniasis is widely used to study the effector functions of T helper cell subsets* in vivo. *Healing and nonhealing* Leishmania major *infections have been correlated with T helper 1 and T helper 2 responses, respectively. In the present study, we determined T cell effector functions* ex vivo, *without any further restimulation and compared them to those obtained following antigen-specific restimulation* in vitro. *Our results show that T helper cell responses are significantly less polarized when determined* ex vivo *as compared to those measured after restimulation* in vitro. *Moreover, the differences in CD4^+^ T cell proliferation observed between healer and nonhealer strains of mice differed* ex vivo *and* in vitro. *Our results suggest that determination of both* ex vivo *as well as* in vitro *T cell responses is crucial to characterize immune responses during experimental leishmaniasis.*

## INTRODUCTION

The leishmaniases are a group of vector-borne parasitic diseases that inflict an immense toll in the developing world; they are major causes of morbidity and mortality and impede economic development. Currently, these diseases affect an estimated 12 million people in 88 countries, and approximately 350 million people are at risk. The leishmaniases belong to the most neglected tropical diseases, affecting the poorest populations, for whom access to diagnosis and effective treatment are most difficult (http://www.who.int/leishmaniasis/en/). Leishmaniases present with a wide range of symptoms, ranging from the self healing cutaneous form, which produces localized skin ulcers; to the mucocutaneous form, which leads to the destruction of mucous membranes of the mouth, throat, nose and neighbouring tissue; to the visceral form, the most severe form of leishmaniasis, in which the mortality rate can be as high at 100%. A multitude of factors, including parasite and vector species, host immune responses, genetic and environmental factors influence the outcome of infection.

T lymphocytes are central players in the host response of all forms of leishmaniasis; they are decisive for both healing and the generation of protective immunity as well as for nonhealing persistent disease and pathology. Experimental studies in inbred strains of mice with *Leishmania (L.) major* mimic the healing and nonhealing manifestation of leishmaniasis and have established the current paradigm of T helper (Th) cell subset involvement (1–3). In this model, the majority of inbred strains of mice develop small lesions that will spontaneously heal within a few weeks; this ability to control infection is associated with the expansion of Th1 cells, characterized by the production of IFN-γ. In contrast, a few strains of mice such as BALB/c develop nonhealing disease, attributed to the expansion of Th2 cells and the production of their signature cytokines interleukin (IL)-4 and IL-13.

*Leishmania* are obligate intracellular parasites in their mammalian host. After transmission into their host, they invade and parasitize mainly macrophages, which are decisive effector cells that either kill or host the intracellular parasites depending on the balance between Th1 and Th2 cytokines: Th1 cytokines induce classical activation of macrophages and inducible nitric oxide synthase (iNOS) that oxidizes l-arginine into nitric oxide (NO), a metabolite responsible for the killing of intracellular *Leishmania* parasites (4). Th2 cytokines result in alternative activation of macrophages and the induction of arginase (5), which metabolizes l-arginine into urea and ornithine; the latter is the main intracellular source for the synthesis of polyamines necessary for parasite growth (6,7).

In addition to the canonical type 1 and type 2 T helper cell responses, IL-10, a cytokine originally described as a type 2 cytokine (8), plays an important role in leishmaniasis. Both CD4^+^CD25^+^FoxP3^+^ T cells (9) and CD4^+^CD25^−^FoxP3^−^ Th1 cells (10) have been shown to express IL-10 and play a crucial role in parasite persistence and in immunosuppression.

In the experimental model of leishmaniasis, the type of immune responses that is associated with healing or nonhealing has been largely defined by measuring cytokine production following *in vitro* restimulation of lymphoid cells with *Leishmania* parasites. However, these do not necessarily reflect immune responses in the infected host *in vivo*. Therefore, to assess immune responses induced in response to infection with *L. major in vivo*, we measured CD4^+^ T cell effector functions directly *ex vivo*, in freshly isolated cells from the lymph nodes draining the cutaneous lesions and compared them to recall responses obtained after *in vitro* restimulation.

## MATERIALS AND METHODS

### Mice

Female BALB/c and CBA mice (6- to 8-week old) (Charles River, UK) were kept in individually vented cages. Animal colonies, screened regularly for mouse pathogens, consistently tested negative. Animal experiments were performed in accordance with Governmental (Home Office) and Institutional Guidelines.

### Experimental infection with *L. major* parasites

For infections, 2 × 10^6^ stationary phase *L. major* LV39 (MRHO/SU/59/P-strain) promastigotes were injected subcutaneously (s.c.) into the footpad (11).

### Proliferation assay

#### Preparation of cells

*Ex vivo*: *L. major* infected mice were treated with 1 mg 5-bromo-2-deoxyuridine (BrdU, Sigma) i.p. once a day for the last 4 days before experiments were terminated. Draining lymph nodes from individual mice were homogenized into single cell suspensions using cell dissociation sieves and labelled as described below.

*In vitro*: Popliteal lymph nodes from 2 or 4 week infected nonhealer (BALB/c) and healer (CBA) strains of mice were homogenized and 5 × 10^6^/mL cells were restimulated for 5 days with 1 × 10^6^ live *L. major* parasites or 1 × 10^6^ crude *L. major* antigen (frozen and thawed three times) in Dulbecco's Modified Eagle's Medium (DMEM) supplemented with 5% foetal bovine serum (FBS), 50 IU/mL penicillin, 50 mg/mL streptomycin and 292 mg/mL l-glutamine (Gibco). BrdU was added to the cell culture 18 h before the cells were harvested and labelled as described below.

Before surface labelling with anti-CD4 mAb (clone H129·19 or GK1·5, Pharmingen), cells were preincubated with 1 µg of rat anti-mouse monoclonal antibody CD32/CD16 (FcγII/III receptor, Pharmingen). Cells were washed, fixed and permeabilized using the method described in (12). Detection of CD4^+^ BrdU^+^ cells was performed using a FACSCalibur (BD) and data were analysed using Summit v4·3 software. Isotype control: 0·57%.

### Intracellular cytokine determination

#### Preparation of cells

*Ex vivo*: Individual draining lymph nodes were harvested from 2 week infected mice and were homogenized into single cell suspensions using cell dissociation sieves.

*In vitro*: Lymph node cells were stimulated with *L. major* parasites as described above and harvested after 5 days.

Cells (1 × 10^6^) were stimulated with 50 ng of phorbol 12-myristate 13-acetate (PMA; Sigma) and 500 ng of ionomycin (Calbiochem) or, as a control, in the presence of complete medium alone for 4 h, with 10 µg of Brefeldin A (Sigma) added for the last 2 h. Before surface labelling with anti-CD4 mAb (clone H129·19 or GK1·5, Pharmingen), cells were preincubated with 1 µg of rat antimouse monoclonal antibody CD32/CD16 (FcγII/III receptor, Pharmingen). Cells were washed, fixed and permeabilized as described in (13) before the anti-cytokine antibodies or the isotype controls were added [anti-IL-4 mAb, clone BVD4–1D11; anti-IFN-γ mAb, clone XMG1·2; anti-IL-10 mAb, clone JES5–16E3; appropriately labelled rat immunoglobulin (Pharmingen)]. Detection of intracellular cytokines was performed using a FACSCalibur (Becton–Dickinson) and data were analysed using Summit v4·3 software.

Isotype control for IFN-γ: 0·27 ± 0·04%, IL-4: 0·25 ± 0·05% and IL-10: 0·22 ± 0·05%, respectively. All cytokines were below the detection limit when the cells were stimulated in the absence of PMA/ionomycin stimulation. The detection limits were defined as percentage isotype control +3 standard deviations: IFN-γ = 0·39%, IL-4 = 0·40% and IL-10 = 0·37%.

iMFI: The integrated mean fluorescent intensity (iMFI) (14) was obtained by multiplying the percentage of CD4^+^ T cells with the value of the mean fluorescent intensity (MFI) for BrdU or with the value of the MFI for the relevant cytokine.

### Luminex

Lymphocytes were stimulated as described above and 3 days later, supernatants were harvested and IFN-γ and IL-4 were detected simultaneously in each sample by the Luminex-based Multiplexed assay (Luminex 100 System). Data were analysed using STarstation V2·0.

### Statistical analyses

Statistical differences were determined using a two-tailed Mann–Whitney test and differences were considered statistically significant at *P* < 0·05.

## RESULTS

### Production of the cytokines IFN-γ, IL-4 and IL-10 is strongly amplified after restimulation

**Figure 1:**
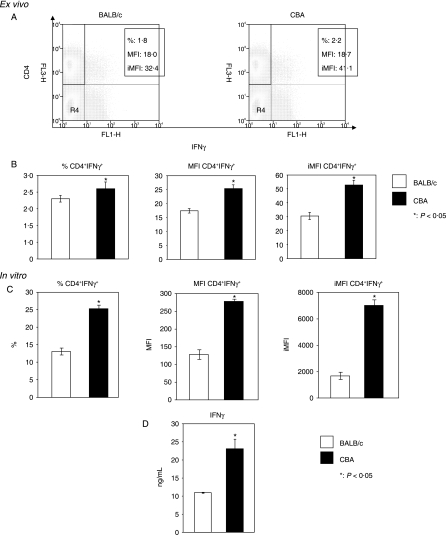
*Ex vivo* and *in vitro* expression of IFN-γ by CD4^+^ T cells. Groups of BALB/c and CBA (*n* = 4) mice were infected with *Leishmania major* parasites in one hind footpad. Two weeks later, individual popliteal lymph nodes were harvested and the percentage of IFN-γ-expressing CD4^+^ T cells was determined directly by flow cytometry: (A) E*x vivo:* Dot plot profiles of CD4^+^IFN-γ^+^ T cells; (B) %, MFI and iMFI of CD4^+^IFN-γ^+^ T cells. (C) *In vitro*: Lymph node cells were restimulated with *L. major* parasites and 5 days later, the percentage of IFN-γ-expressing CD4^+^ T cells was determined by flow cytometry. The error bars represent SD. (D) Lymph node cells were restimulated with *L. major* parasites and 3 days later, the supernatants were harvested and the cytokine content was determined by Luminex. The error bars represent standard deviations. Data show the results of one representative experiment out of five independent experiments.

In the experimental model of leishmaniasis, immune responses have mostly been characterized by restimulating lymphoid cells from infected mice with *Leishmania*antigen *in vitro* (1,2). To determine how closely these responses reflect the host response to *Leishmania* infection, we compared CD4^+^ T cell responses from *L. major* infected mice *ex vivo* and *in vitro*. We infected nonhealer mice (BALB/c) and healer mice (CBA) with *L. major* promastigotes and measured 2 weeks later CD4^+^ T cell effector functions directly *ex vivo* in the draining lymph nodes and compared them with those obtained after *in vitro* restimulation with *L. major* parasites as antigen. We first assessed the production of the type-1 cytokine IFN-γ in *L. major* infected healer and nonhealer strains directly *ex vivo*, without *in vitro* restimulation. As shown in Figure 1A, the frequency of CD4^+^IFN-γ^+^ T cells was lower in the lymph nodes from BALB/c mice as compared to CBA mice (1·8 ± 0·1% vs. 2·1 ± 0·2%, *P* < 0·05, Figure 1A,B). A recent study has described a new metric parameter, the integrated mean fluorescence intensity (iMFI), which reflects more precisely the total functional response of activated T cells (14). iMFI is calculated by multiplying the percentage of positive cells, which represents the magnitude of the response by the MFI, which represents the quality of the response. As shown in Figure 1, the CD4^+^IFN-γ^+^ iMFI was lower in BALB/c than in CBA mice when determined directly *ex vivo*(30·6 ± 2·6 vs. 52·8 ± 3·4, *P* < 0·05). Similar results were obtained when the frequency, MFI and iMFI of CD4^+^IFN-γ^+^ T cell were determined directly *ex vivo* 4 weeks post *L. major* infection (*P <* 0·05, Table 1A).

**Table 1:** Percentage, MFI and iMFI of CD4^+^IFN-γ^+^ T cells

**(A)**			
*Ex vivo*	Percentage of CD4^+^IFN-γ^+^ (%)	MFI CD4^+^IFN-γ^+^	iMFI CD4^+^IFN-γ^+^
BALB/c	1·1 ± 0·2	22·3 ± 2·1	25·0 ± 2·4
CBA	1·6 ± 0·3	23·4 ± 1·9	36·5 ± 2·9

**(B)**			

*In vitro*	Percentage of CD4^+^IFN-γ^+^ (%)	MFI CD4^+^IFN-γ^+^	iMFI CD4^+^IFN-γ^+^

BALB/c	13·2 ± 1·2	225·0 ± 21·0	2970 ± 32·9
CBA	30·3 ± 2·8	398·0 ± 20·9	12059·4 ± 98·7

Groups of BALB/c and CBA (*n* = 4) mice were infected with *L. major* parasites in one hind footpad. Four weeks later, individual popliteal lymph nodes were harvested and the percentage of IFN-γ-expressing CD4^+^ T cells was determined by flow cytometry:

(A) *Ex vivo*: Percentage, MFI and iMFI of CD4^+^IFN-γ^+^ T cells.

(B) *In vitro*: Lymph node cells were restimulated with *L. major* parasites and 5 days later, the percentage of IFN-γ-expressing CD4^+^ T cells was determined by flow cytometry.

Data are ±SD and show the results of one representative experiment out of three independent experiments.

Next we restimulated lymph node cells from 2 week infected mice with *L. major* parasites for 5 days (15) and measured the percentages, MFI and iMFI of CD4^+^IFN-γ^+^. As shown in Figure 1C, after *in vitro* restimulation these three parameters were considerably higher in the lymph node cultures from CBA mice as compared to those of BALB/c mice (*P <* 0·05). Similar results were obtained when we measured IFN-γ levels in the supernatants of restimulated lymph node cells (23·1 ± 2·7 pg/mL vs. 10·9 ± 0·9 pg/mL, *P* < 0·05, Figure 1D). Moreover, a comparable increase in percentage, MFI and iMFI of CD4^+^IFN-γ^+^ T cells was observed when lymphoid cells from 4 weeks infected mice were restimulated with *L. major* parasites *in vitro* (*P <* 0·05, Table 1B).

The higher production of IFN-γ observed *in vitro* with cells from CBA mice (Figure 1C,D) was not due a higher number of cells after restimulation, as the total number of cells after *in vitro* restimulation was even higher in the BALB/c mice (15·0 × 10^5^ ± 0·8 × 10^5^ cells/mL (BALB/c cultures) vs. 8·9 × 10^5^ ± 0·6 × 10^5^ cells/mL (CBA cultures), data not shown).

The results presented in Figure 1 show that by restimulating lymph node cells *in vitro*, the differences in percentage, MFI and iMFI of CD4^+^IFN-γ^+^ T cells between BALB/c and CBA are amplified as compared to *ex vivo*.

**Figure 2:**
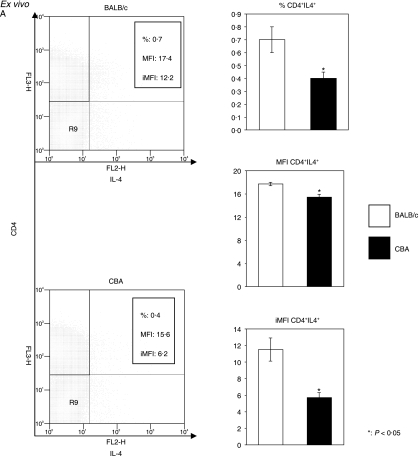

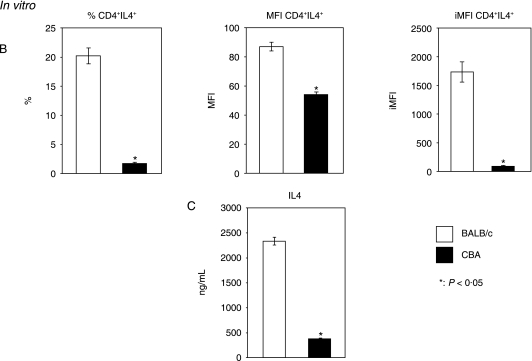
*Ex vivo* and *in vitro* expression of IL-4 by CD4^+^ T cells. Groups of BALB/c and CBA (*n* = 4) mice were infected with *Leishmania major* parasites in one hind footpad. Two weeks later, individual popliteal lymph nodes were harvested and the percentage of IL-4-expressing CD4^+^ T cells was determined directly by flow cytometry: (A) *Ex vivo*: Dot plot profiles of CD4^+^IL-4^+^ T cell and percentage, MFI and iMFI of CD4^+^IL-4 T cells. (B) *In vitro*: Lymph node cells were restimulated with *L. major* parasites and 5 days later, the percentage of IL-4-expressing CD4^+^ T cells was determined by flow cytometry. The error bars represent standard deviations. (c) Lymph node cells were restimulated with *L. major* parasites and three days later, the supernatants were harvested and the cytokine content was determined by Luminex. The error bars represent standard deviations. Data show the results of one representative experiment out of five independent experiments.

Next, we measured the *ex vivo* and *in vitro*production of IL-4 by CD4^+^ T cells in draining lymph nodes of BALB/c and CBA mice two weeks post infection. Production of IL-4 was higher in the BALB/c as compared to CBA mice (% CD4^+^IL-4^+^: 1·8 fold increase, MFI CD4^+^IL-4^+^: 1·1 fold increase, iMFI CD4^+^IL-4^+^: 2·0 fold increase, Figure 2A). Comparable results were obtained *ex vivo* 4 weeks post infection (*P <* 0·05, Table 2A). Similarly to the tendency observed by analysing IFN-γ production, the differences in IL-4 production by CD4^+^ T cells between nonhealer and healer mice were considerably increased when lymph nodes cells were restimulated with *L. major* parasites *in vitro* (% CD4^+^IL-4^+^: 13·2 fold increase, MFI CD4^+^IL-4^+^: 1·6 fold increase, iMFI CD4^+^IL-4^+^: 21·1 fold increase, Figure 2B). The IL-4 levels were also significantly higher in the supernatants of restimulated lymph nodes cells (2330·0 ± 75·1 pg/mL vs. 378·5 ± 16·2 pg/mL, *P* < 0·05, Figure 2C). A similar tendency was observed when lymph node cells from 4 week infected mice were restimulated with *L. major*parasites *in vitro* (*P <* 0·05, Table 2A).

**Table 2:** Percentage, MFI and iMFI of CD4^+^IL-4^+^ T cells

**(A)**			
*Ex vivo*	Percentage of CD4^+^IL-4^+^ (%)	MFI CD4^+^IL-4^+^	iMFI CD4^+^IL-4^+^
BALB/c	1·3 ± 0·2	25·2 ± 2·1	31·5 ± 2·7
CBA	0·7 ± 0·1	21·9 ± 1·9	14·9 ± 1·1

**(B)**			

*In vitro*	Percentage of CD4^+^IL-4^+^ (%)	MFI CD4^+^IL-4^+^	iMFI CD4^+^IL-4^+^

BALB/c	14·2 ± 1·2	125·0 ± 11·2	1775·0 ± 121·9
CBA	3·3 ± 0·2	82·1 ± 4·6	270·9 ± 15·6

Groups of BALB/c and CBA (*n* = 4) mice were infected with *L. major* parasites in one hind footpad. Four weeks later, individual popliteal lymph nodes were harvested and the percentage of IL-4-expressing CD4^+^ T cells was determined by flow cytometry: (A) *Ex vivo*: Percentage, MFI and iMFI of CD4^+^IL-4^+^ T cells.

(B) *In vitro*: Lymph node cells were restimulated with *L. major* parasites and 5 days later, the percentage of IL-4-expressing CD4^+^ T cells was determined by flow cytometry.

Data are ±SD and show the results of one representative experiment out of three independent experiments.

**Figure 3:**
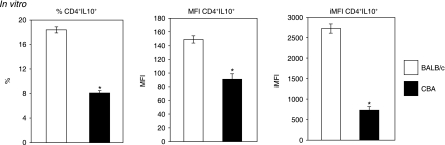
*In vitro* expression of IL-10 by CD4^+^ T cells. Groups of BALB/c and CBA (*n* = 4) mice were infected with *L. major* parasites in one hind footpad. Two weeks later, individual popliteal lymph nodes were harvested and lymph node cells were restimulated with *L. major* parasites and 5 days later, percentage, MFI and iMFI of IL-10-expressing CD4^+^ T cells was determined by flow cytometry. The error bars represent standard deviations. Data show the results of one representative experiment out of five independent experiments.

The regulatory cytokine IL-10 could not be detected directly *ex vivo* as it was below detection limit both 2 and 4 weeks post infection (detection limit: 0·37%, data not shown). However, following *in vitro* restimulation, IL-10 production was clearly detectable and the percentage, MFI and iMFI were all significantly increased in the lymph nodes cells from the BALB/c mice as compared to CBA mice (Figure 3A.*P* < 0·05). Similar results were obtained when lymphoid cells from 4 week infected BALB/c and CBA mice were restimulated with *L. major* parasites *in vitro* (*P <* 0·05, Table 3).

**Table 3:** Percentage, MFI and iMFI of CD4^+^IL-10^+^

*In vitro*	Percentage of CD4^+^IL-10^+^ (%)	MFI CD4^+^IL-10^+^	iMFI CD4^+^IL-10^+^
BALB/c	14·2 ± 1·2	125·0 ± 11·2	1775·0 ± 121·9
CBA	3·3 ± 0·2	82·1 ± 4·6	270·9 ± 15·6

Groups of BALB/c and CBA (*n* = 4) mice were infected with *L. major* parasites in one hind footpad. Four weeks later, individual popliteal lymph nodes were harvested restimulated with *L. major* parasites and 5 days later, percentage, MFI and iMFI of IL-10-expressing CD4^+^ T cells was determined by flow cytometry.

Data are ±SD and show the results of one representative experiment out of three independent experiments.

To determine how different preparations of *L. major* parasites might affect the levels of cytokine production, we stimulated lymph node cells from two weeks infected BALB/c mice and CBA mice with live *L. major* parasites or with crude *L. major* antigen (freeze and thawed *L. major* parasites). As shown in Table 4, the different antigenic preparation did not affects the levels of IFN-γ, IL-4 or IL-10 produced.

**Table 4:** Cytokine production following *in vitro* restimulation with different preparations of *L. major* antigens

BALB/c mice	iMFI CD4^+^IFN-γ^+^	iMFI CD4^+^IL-4^+^	iMFI CD4^+^IL-10^+^
Live *L. major*	1297·1 ± 112·0	985·6 ± 85·2	2926·0 ± 157·5
Crude *L. major* antigen	1189·2 ± 98·6	1023 ± 96·3	2786·3 ± 256·7

CBA mice	iMFI CD4^+^IFN-γ^+^	iMFI CD4^+^IL-4^+^	iMFI CD4^+^IL-10^+^

Live *L. major*	8521·3 ± 612·0	125·6 ± 9·6	826·9 ± 74·2
Crude *L. major* antigen	9146·8 ± 529·4	142·5 ± 8·3	901·3 ± 85·7

Groups of BALB/c and CBA (*n* = 4) mice were infected with *L. major* parasites in one hind footpad. Two weeks later, individual popliteal lymph nodes were harvested and restimulated with live *L. major* parasites or crude *L. major* antigen preparation. Five days later, the iMFI of cytokine-expressing CD4^+^ T cells was determined by flow cytometry.

Data are ± standard deviations and show the results of one representative experiment out of two independent experiments.

### The proliferative response of CD4^+^ T cells from *L. major* infected healer and nonhealer strains of mice differs both *ex vivo* and *in vitro.*

**Figure 4:**
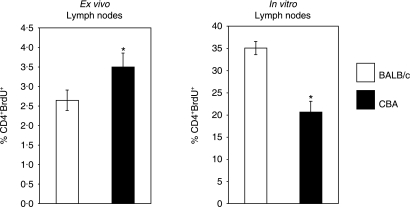
*Ex vivo* and *in vitro* CD4^+^ T cells proliferation. Groups of BALB/c and CBA (*n* = 4) mice were infected with *L. major* parasites in one hind footpad and were treated with 1 mg BrdU (Sigma) i.p. once a day for the last 4 days before experiments were terminated. Individual popliteal lymph nodes were harvested and the percentage of BrdU^+^CD4^+^ T cells was determined directly *ex vivo* by flow cytometry or following restimulation of lymph node cells with *L. major* parasites for 5 days. The error bars represent standard deviations.

To characterize further CD4^+^ T cell effector functions *ex vivo* and *in vitro* in healer and nonhealer strains of mice, we measured the capacity of CD4^+^ T cells to proliferate. We first measured *L. major*-induced proliferation of CD4^+^ T cells directly *ex vivo* by quantifying the frequency of CD4^+^ T cells that had incorporated BrdU *in vivo*. We found that there was a small decrease in the frequency of proliferating CD4^+^ T cells in the draining lymph nodes of nonhealer mice compared with healer mice 2 weeks post-infection (2·65 ± 0·26% vs. 3·50 ± 0·36%, *P* < 0·05, Figure 4). Similar results were obtained 4 weeks post-infection (Table 5A). In contrast, after restimulation *in vitro* with *L. major* antigens, CD4^+^ T cells from nonhealer mice proliferated more efficiently then those of healer mice (35·1 ± 1·5% vs. 20·6 ± 2·4%, Figure 4). A similar tendency was observed 4 weeks post-infection (*P <* 0·05, Table 5B).

**Table 5:** % of CD4^+^BrdU^+^ T cells

**(A)**	
*Ex vivo*	Percentage of CD4^+^BrdU^+^ (%)
BALB/c	1·5 ± 0·2
CBA	1·9 ± 0·1
**(B)**	
*In vitro*	Percentage of CD4^+^BrdU^+^ (%)
BALB/c	38·5 ± 2·9
CBA	28·8 ± 4·2

Groups of BALB/c and CBA (*n* = 4) mice were infected with *L. major* parasites in one hind footpad and were treated with 1 mg BrdU (Sigma) i.p. once a day for the last 4 days before experiments were terminated. Individual popliteal lymph nodes were harvested and the percentage of BrdU^+^CD4^+^ T cells was determined directly *ex vivo* (A) by flow cytometry or following restimulation of lymph node cells *in vitro* (B) with *L. major* parasites for 5 days later.

Data are ±SD and show the results of one representative experiment out of three independent experiments.

These results show that the *ex vivo* frequency of proliferating CD4^+^ T cells was lower in the draining lymph nodes of nonhealer mice than in those of healer mice. However, CD4^+^ T cells from draining lymph nodes of nonhealer mice displayed a more pronounced proliferative response to restimulation with *Leishmania* than those from healer mice.

## DISCUSSION

Infections of mice with different strains of *Leishmania* parasites have proved to be very useful models to characterize the development and regulation of immune responses during leishmaniasis. The balance of cytokines produced by Th1 and Th2 cells generated in response to *L. major* infection provides an important conceptual framework for understanding healing vs. nonhealing forms of leishmaniasis. Healing of infection and development of protection against leishmaniasis crucially depends on the development of Th1 type immunity that can instruct parasitical control mechanisms in infected macrophages. However, the mechanisms accounting for insufficient control of growth and dissemination of *Leishmania*parasites and resulting in persistent nonhealing disease in infected patients are less well understood (16).

Studies with *L. major* parasites in particular have clearly established the current Th1/Th2 paradigm in which control of infection is associated with the expansion of Th1 cells, characterized by the production of IFN-γ and the use of gene knockout mice confirmed the important role of Th1 type responses in the control of disease (17–19). In contrast, nonhealing disease has been attributed to the expansion of Th2 cells, characterized by the production of IL-4 (1–3). In this context, it is important to note that Th2 cells and the spectrum of cytokines they release is not only impacting on the immune response, they will directly promote the growth of the intracellular parasites by inducing arginase 1, which catalyses the hydrolysis of l-arginine into urea and the amino acid l-ornithine; the latter is the first building block for the generation of polyamines which are essential for the intracellular growth of *Leishmania* parasites (6,7). The regulation of immune responses against *Leishmania* parasites is complex and Th2 dominance cannot fully explain nonhealing (20–24); in addition, intralesional injection of IL-4 has been shown to induce healing and immunity to reinfection (25). IL-4 can instruct dendritic cells to produce IL-12 and promote Th1 development and healing in BALB/c mice (26). Furthermore, it has been shown that cure can be achieved in an IFN-γ independent manner (18,27). Moreover, in human leishmaniasis, the different clinical outcomes do not appear to be associated with polarized Th1- or Th2-type responses: whereas IL-4 is higher in the plasma of patients with visceral leishmaniasis (VL) (28,29), a number of proinflammatory cytokines such as IL-1, IL-6, IL-12, IFN-γ and TNF-α are also increased (30,31).

Our *in vitro* results, in agreement with those of many others [reviewed in (1–3)], substantiate this Th1/Th2 model: we show that after *in vitro* restimulation, lymphoid cells from healer mice display a strong IFN-γ response, whereas in nonhealer mice there is predominantly an IL-4 response. However, when Th responses were measured directly *ex vivo*, without any further restimulation, we show that the dichotomy of Th cell responses is not as pronounced as those after *in vitro* restimulation. These results suggest that the healing or nonhealing phenotype of *L. major* infected mice does not correlate with such a polarized Th1 or Th2 response when determined *ex vivo*. The causes for this strong increase in the frequencies of CD4^+^IFN-γ^+^ or CD4^+^IL-4^+^ T cells following *in vitro* restimulation are unclear. We can exclude that apoptosis was more prominent in any of the cultures, since there was no increase in caspase^+^ CD4^+^ T cells (P. Kropf, unpublished results). It is possible that factors such as costimulation or antigen presentation (32) that are specifically influenced by *in vitro* restimulation conditions influence this strong preferential expansion of Th1 or Th2 cells. Furthermore, it is possible that flow cytometry is not sensitive enough to detect CD4^+^ T cells that express low levels of IL-4 and IL-10. Indeed, IL-10 was below detection limit when determined *ex vivo* even though it was clearly detectable following *in vitro* restimulation with *L. major* parasites. IL-10 mRNA has been previously measured in the draining lymph node of healer and nonhealer mice following infection with *L. major* parasites (33,34). Taken together, these results suggest that flow cytometry might not be a technique that is sensitive enough to measure the frequency of CD4^+^IL-10^+^ T cells directly *ex vivo* without any further restimulation. The production of IL-10 by CD4^+^ T cells was clearly detectable by flow cytometry following *in vitro* restimulation with *L. major* parasites; CD4^+^ T cells from nonhealer mice produce significantly more IL-10 than those from healer strains of mice. It is well established that IL-10 has a down-regulatory effect on macrophages and can inhibit IFN-γ-induced macrophage activation (35). Moreover, IL-10 synergizes with IL-4 in the induction of alternative activation of macrophages and our previous results clearly demonstrated that IL-10 and IL-4 synergize in promoting the parasite growth via enhanced arginase induction and polyamine synthesis (7). Recent work indicates that failure to control leishmaniasis is not due to a dominant Th2 or a defective Th1 response *per se* but could be ascribed to a concomitant production of IL-10 (36). Moreover, experimental evidence showed that IL-10 deficient BALB/c mice as well as inhibition of binding of IL-10 to its receptor improved the control of *L. major*infection in nonhealer strains of mice (37,38). Interestingly, some *L. major* isolates produce nonhealing lesions in strains of mice such as C57BL/6 that are usually considered to be healers and mount a Th1 response, thus, this work clearly shows that although Th1 responses are required for healing, they are not sufficient (24). Importantly, our *in vitro* results as well as those from others underpin these results as they show that IL-10 is strongly expressed after restimulation of lymphoid cells from nonhealer mice as compared to healer mice and strengthen further the role that IL-10 might have in the pathogenesis of nonhealing leishmaniasis.

It is also important to note that the experimental model of infection that has been used to characterize Th cell responses does not reproduce the biology of natural infection; indeed, factors such as the dose of parasites inoculated (39–41), the site of infection (39,42), the presence of saliva (43) or parasite components, such as filamentous proteophosphoglycan (40) have been shown to greatly influence the outcome of infection. Little is known about T cell responses in mice directly infected with sandflies and more studies in the natural infection model are of paramount importance to fully understand T cell polarization and effector functions.

To characterize further T cell effector functions, we determined the proliferation of CD4^+^ T cells *ex vivo* or after restimulation *in vitro*: there was a higher frequency of CD4^+^BrdU^+^ T cells in CBA mice when measured *ex vivo*, however, after *in vitro* restimulation, the frequency of CD4^+^BrdU^+^ T cells was significantly higher in BALB/c mice as compared to CBA mice. It is possible that the culture and restimulation conditions favour a higher activation of CD4^+^ T cells from BALB/c mice; indeed, the frequency of CD4^+^CD25^+^, CD4^+^CD40L^+^, CD4^+^OX40L^+^ and CD4^+^CD62L^−^ T cells were significantly higher in the cultures of BALB/c mice as compared to CBA mice (P. Kropf, unpublished results).

Characterization of T helper cell responses in healer and nonhealer strains of mice has been instrumental in our understanding of the mechanisms resulting in healing and nonhealing leishmaniasis. Our results suggest that determining both *ex vivo* and *in vitro* T cell effector functions are important to dissect and understand the development and regulation of immune responses during experimental leishmaniasis.
